# Trends in Hospitalizations of Patients with Hepatitis C Virus in Poland between 2012 and 2022

**DOI:** 10.3390/jcm13185618

**Published:** 2024-09-22

**Authors:** Agnieszka Genowska, Dorota Zarębska-Michaluk, Krystyna Dobrowolska, Krzysztof Kanecki, Paweł Goryński, Piotr Tyszko, Katarzyna Lewtak, Piotr Rzymski, Robert Flisiak

**Affiliations:** 1 Department of Public Health, Medical University of Bialystok, 15-295 Bialystok, Poland; agnieszka.genowska@umb.edu.pl; 2 Department of Infectious Diseases and Allergology, Jan Kochanowski University, 25-317 Kielce, Poland; dorota1010@tlen.pl; 3 Collegium Medicum, Jan Kochanowski University, 25-317 Kielce, Poland; 4 Department of Social Medicine and Public Health, Medical University of Warsaw, 02-091 Warsaw, Poland; krzysztof.kanecki@wum.edu.pl (K.K.); piotr.tyszko@wum.edu.pl (P.T.); katarzyna.lewtak@wum.edu.pl (K.L.); 5 Department of Population Health Monitoring and Analysis, National Institute of Public Health NIH-National Research Institute, 00-791 Warsaw, Poland; pawel@pzh.gov.pl; 6 Institute of Rural Health, 20-090 Lublin, Poland; 7 Department of Environmental Medicine, Poznan University of Medical Sciences, 60-806 Poznan, Poland; rzymskipiotr@ump.edu.pl; 8 Department of Infectious Diseases and Hepatology, Medical University of Bialystok, 15-540 Bialystok, Poland; robert.flisiak1@gmail.com

**Keywords:** hepatitis C, comorbidities, epidemiology, hospitalizations, length of stay

## Abstract

**Background:** Analyzing hospitalizations of patients with hepatitis C virus (HCV) infection is essential for an effective action plan to eliminate hepatitis C as a public health threat. This study aimed to explore trends in hospitalizations of patients with HCV infection and factors related to these hospitalizations. **Methods:** This 11-year retrospective study (2012–2022) explored trends in hospitalizations of patients with HCV infection in Poland based on data from the Nationwide General Hospital Morbidity Study. **Results**: The mean age of individuals was 55 years, with hospitalization rates among men and women of 15.5 and 13.7 per 100,000 population, respectively. Hospitalizations were 1.8-fold higher among urban residents. The most frequent comorbidities were digestive (24%) and cardiovascular (18%) diseases. During the studied period, the hospitalization rates significantly decreased from 31.9 per 100,000 in 2012 to 5.0 per 100,000 in 2022, with stays requiring 0–3, 4–7, and ≥8 days becoming 8-fold, 6-fold, and 4-fold less frequent, respectively. The flattening of hospitalizations was apparent across all age groups, including children. **Conclusions**: While significant progress has been made in managing HCV in Poland, continued efforts are required to eliminate disparities in care and to sustain the momentum toward HCV elimination, particularly through enhanced political commitment and the implementation of comprehensive national screening programs.

## 1. Introduction

Based on recent World Health Organization (WHO) estimates, 50 million people are living with hepatitis C virus (HCV) infection globally, and only 36% of them are aware of this fact [1]. Despite years of efforts, preventive vaccines remain unavailable [2,3]. It is estimated that approximately one-third of HCV-infected individuals can spontaneously clear the virus within 12 months due to genetic and immunologic factors, with the remaining patients developing a chronic infection [4,5,6]. Its higher incidence has been observed among people born between 1946 and 1964, the so-called “Baby Boomers”, and more recently among young people due to the ongoing opioid crisis in selected regions of the world [7,8]. The bimodal age distribution prompted a change in US Centers for Disease Control and Prevention (CDC) guidelines toward universal screening of all adults at least once in their lifetime, regardless of risk factors. At the same time, at-risk individuals continuing risky behavior should be screened more frequently [9,10]. Such an approach is pivotal in introducing the treatment as early as possible and decreasing the burden of HCV infection on individual health as well as the economic costs of healthcare [11,12,13]. 

Per the guidelines of the CDC and national scientific societies, universal testing of pregnant women is also recommended since its detection and treatment are also important for the health of newborns [14,15]. Some countries have already put these recommendations into practice with universal testing of adult citizens, an example being Lithuania, where a national screening program has been introduced, resulting in 44% of the target population being screened within the first year, with positive results for anti-HCV antibodies documented in 1.5% of persons [16,17]. In neighboring Poland, despite years of efforts from scientific societies, such a universal screening program has not been implemented, which is one of the reasons why reaching the WHO’s goal of eliminating HCV infection as a public health by 2030 is unrealistic [18]. 

However, based on available data, the prevalence of HCV infection in the adult population in Poland is estimated to be less than 1%, with viremic cases accounting for less than half of the total, translating to about 140,000 infected individuals, of which 31% are aware of the disease [15,19]. A recent analysis showed that between 2009 and 2021, the number of newly diagnosed HCV infections exceeded 36,000, and a total of more than 2000 HCV-related deaths were recorded [20]. 

Patients with chronic HCV infection report lower quality of life and a significant negative impact on daily functioning at home and work through reduced productivity, increased absence, and higher costs of health care services [21,22,23]. However, the public health burden associated with HCV is largely due to the most severe complications of the disease, such as liver cirrhosis, which is related to the risk of decompensation, bleeding from esophageal varices, and the development of hepatocellular carcinoma [24,25,26]. The introduction of safe and highly effective interferon-free therapies based on direct-acting antivirals (DAA) has significantly improved the prognosis of HCV-infected patients, including those in the end-stage of the disease, decreased their cardiovascular risk, and benefited the quality of life of treated patients [27,28,29]. 

Nevertheless, persons with HCV infection still undergo diagnostic tests to assess the severity of liver disease, and those with cirrhosis, even after successful DAA therapy, require medical surveillance and hospital intervention due to the advancement of liver disease [30,31,32,33]. Data related to the need for hospitalization in patients with HCV infection, both related to diagnostics and therapeutic interventions, are necessary to create an effective action plan to eliminate HCV as a public health threat [34,35,36]. Therefore, the present study aimed to analyze trends in hospitalizations of patients with HCV infection in Poland from 2012 to 2022, taking into account the length of hospital stays and exploring factors related to these hospitalizations. 

## 2. Materials and Methods

In this study, a retrospective, population-based analysis of data from the Nationwide General Hospital Morbidity Study gathered by the National Institute of Public Health NIH—National Research Institute in Poland was performed. All hospitals, except psychiatric ones, submitted data on each case of hospitalization as part of the Programme of Statistical Surveys of Official Statistics in Poland. The data on each case of hospitalization included information on diagnosed diseases (in the form of ICD-10 codes), dates of admission and discharge, discharge status (including death and causes of death), sex, date of birth, and place of residence. The scope and format of the information are precisely defined in the MZ-Szp11 case form. Since 2000, the survey has covered all hospital admissions, and the data transfer has been conducted through a dedicated IT system. These data are also submitted to WHO, Organization for Economic Co-operation and Developmen, and Eurostat via Statistic Poland. Detailed information about the survey is available at medstat.waw.pl. These data were anonymized and did not allow for patient identification. All first-time hospital admissions between 2012 and 2022, whose primary reason for admission was coded B18.2 (chronic viral hepatitis C)—59,949 cases and B17.1 (acute hepatitis C)—1621 cases, were initially included in the analysis. It should be noted that, especially in the first year of the study, some cases may represent cases of subsequent hospitalizations. However, this phenomenon is reduced by the long follow-up time. 

After verifying the completeness of the data, 170 cases were excluded from further analysis, representing 0.28% of the total dataset. The reasons for exclusion were missing data: place of residence—162 cases, age–5 cases, the length of stay (LOS)—3 cases. Data on the demographic structure of the Polish population were obtained from publicly available materials from Statistics Poland [37]. 

Patients were categorized into three groups based on the LOS. Hospitalizations lasting 1–3 days and 4–7 days mainly included patients undergoing the qualification process before treatment initiation. LOS lasting ≥8 days concerned patients needing hospital treatment; as such, the reimbursement from the National Health Fund in this case was appropriately higher. 

The calculated data included (1) the number and % of first-time hospitalizations and rates per 100,000 population per year: total, by sex, and place of residence for 2012–2020, (2) rates of first-time hospitalizations per 100,000 population per year by sex and place of residence for 2012–2022 in total and by year in LOS classes (0–3 days, 4–7 days, ≥8 days), (3) mean and median age of hospitalizations in 2012–2022 (total) and by year in LOS classes, (4) mean rates of first-time hospitalizations per 100,000 population per year by age in LOS classes, and (5) per 100,000 ratio men/women and urban/rural. 

The Statistica version 13 package (StatSoft Inc., Tulsa, OK, USA) was used to analyze the data. The chi-square test of independence was used. A *p*-value of less than 0.05 was considered statistically significant.

The study was approved by the Ethics Committee of the Medical University of Bialystok (approval number APK-002-149-2024, date of approval 22 February 2024.

## 3. Results

The number of first-time hospital admissions decreased from 12,312 in 2012 to 1337 in 2021, with an increase to 1882 in 2022 (Table 1).

Among the 61,400 hospitalized patients with acute or chronic hepatitis C, 51.6% were men (1.14 men-to-women hospitalization ratio) and 72.2% inhabited urban areas (1.81 urban-to-rural residency). 

Table 2 shows data on the sex and place of residence of the hospitalized patients in relation to LOS classes. A slight excess of male vs. female hospitalizations per 100,000 in classes 0–3 (9.84 vs. 9.34) and ≥8 LOS classes (2.71 vs. 2.44) was observed, and a large one in LOS classes 4–7 (2.99 vs. 1.87). There was a clear advantage of the predominance of urban residents over rural residents in all LOS classes.

The most significant number of hospitalizations required 0–3 day LOS (*n* = 40,399; 65.8%; 9.58/100,000). The number of hospitalizations in the other two LOS categories (4–7 days and ≥8 days) was lower, and no significant differences were observed among them (*n* = 10,173 vs. 10,828; 16.6% vs. 17.6%; 2.41/100,000 vs. 2.57/100,000, respectively). Table 3 summarizes the changes in hospitalization rates per 100,000 from 2012 to 2022 across different LOS classes. 

**Figure 1:**
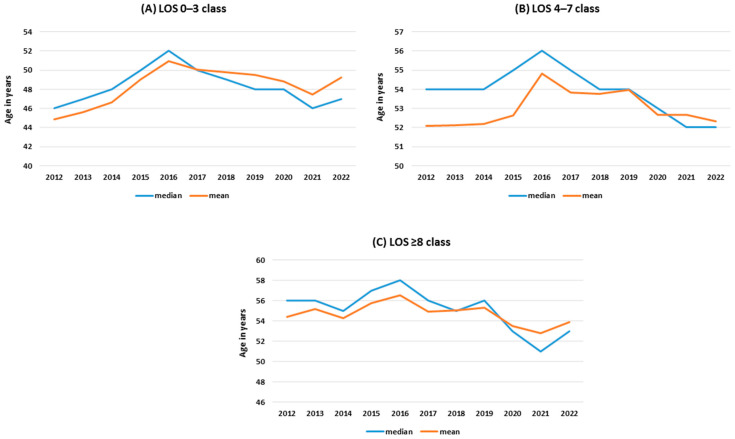
Mean and median age of hospitalized patients in different length of stay (LOS) classes.

**Figure 2:**
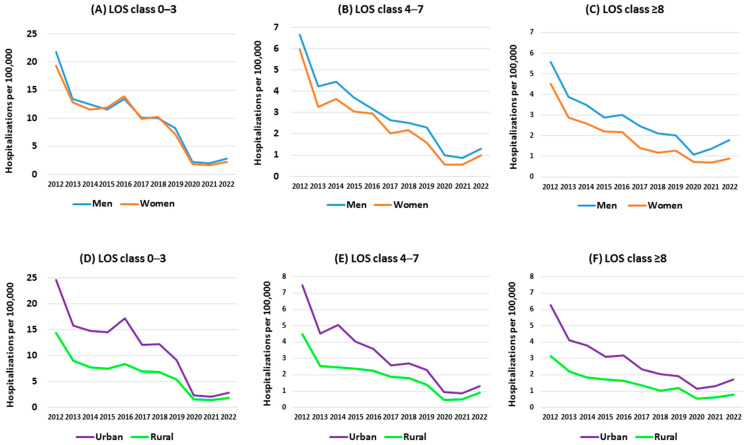
Hospitalizations per 100,000 by sex (**A**–**C**) and place of residence (**D**–**F**) in different length of stay (LOS) classes.

The average age of those hospitalized in 2012-2022 was 54.9, with the lowest in 2021 at 52.8 and the highest in 2016 at 56.6 (t = 4.55, *p* < 0.05). The median age values were 56–58–51 years, respectively. Changes in mean and median age over the analyzed period in LOS classes are shown in Figure 1A–C. In the LOS 0–3 and ≥8 classes, an increase in age measures of hospitalized patients was observed after 2021. Hospitalization rates in LOS class 0–3 decreased between 2012 and 2020 and showed a slight increase between 2021 and 2022. The phenomenon was characterized by much lower dynamics in the other LOS classes, and similarly in the male and female groups (Figure 2A–C). An analogous phenomenon occurred in the hospitalizations of urban and rural residents, with the dynamics of change being more significant in the case of hospitalizations of urban residents (Figure 2D–F). 

**Figure 3:**
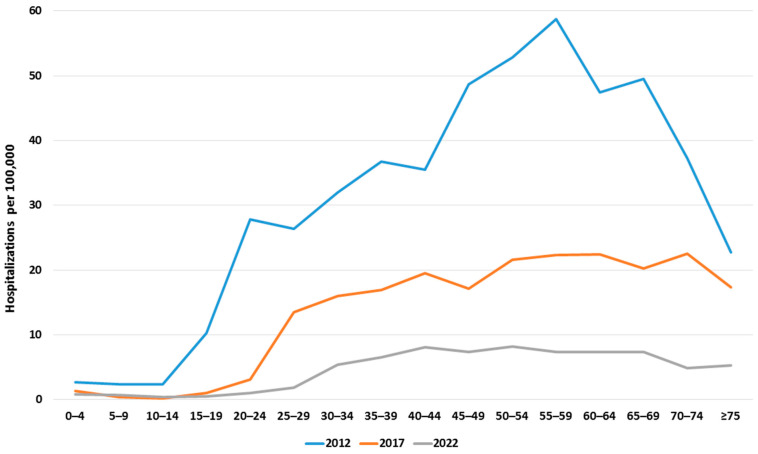
Hospitalizations per 100,000 in age classes in selected years.

Regardless of age and sex, the rate of hospitalizations in all LOS classes was the lowest in 2020, a year when SARS-CoV-2 started to spread globally and the COVID-19 pandemic was declared. The intensity of hospitalization was also analyzed by patient age classes at the initial, middle, and final year of follow-up, as shown in Figure 3. The figure reveals two phenomena. The first is a shift in the starting point of the noticeable increase in hospitalizations per 100,000 population from the age class 10–14 in 2012, from 15–19 in 2017, and from 20–24 in 2022. In 2012, the number of hospitalizations per 100,000 increased from 2.37 in the 10–14 age class to 10.28 in the 15–19 age class, and in 2017 and 2022, respectively, from 1.0 in the 15–19 age class to 3.11 in the 20–24 age class and from 1.02 in the 25–29 age class to 1.84 in the 30–34 age class. The second observed phenomenon was that the rate of hospitalizations was flattened in all age groups, including children.

In the study group, a total of 50,216 diagnoses of comorbidities were reported, of which 20,648 were in the LOS 0–3 class, and 13,245 and 16,323 were in the LOS 4–7 and LOS ≥8 classes, respectively. The most frequently reported comorbidities were diseases of the digestive system (23.50%), diseases of the circulatory system (18.04%), diseases of the blood and blood-forming organs, certain disorders involving the immune mechanism (12.42%), and endocrine, nutritional, and metabolic diseases (11.86%). The distribution of reporting of comorbidities in the LOS classes is shown in Table 4.

Diseases of the digestive system and the D50–D89 group were most frequently reported in LOS classes 4–7, while diseases of the circulatory system, and endocrine, nutritional, and metabolic diseases were most frequently reported in LOS classes 0–3. In the latter group, a decrease in reporting related to the length of hospital stay is evident. With the exception of the neoplasms and diseases of the genitourinary system groups, all differences in the frequency of reporting of comorbidities in the LOS classes were statistically significant (*p* < 0.05). 

## 4. Discussion

The present study provides updated data on hospitalizations among individuals with hepatitis C in Poland, enabling their characterization and understanding of how their profile changed over the course of 11 years. During this period, the situation of HCV-infected patients evolved due to improved access to diagnostics and treatment and the introduction and availability of DAAs, including pangenotypic regimes [38]. Our analysis highlights the simultaneous changes in the rate of hospitalizations due to hepatitis C, which has significantly decreased between 2012 and 2022 (from 31.91 per 100,000 in 2012 to 4.95 per 100,000 in 2022), including those that required extended stays. This indirectly indicates the diminished HCV burden in Poland, though it has been previously concluded that this effect could be much more profound under improved political commitment to the elimination of HCV and the implementation of national screening programs [19,39].

It is well established that HCV infection in Europe remains more prevalent in men, with a male-to-female ratio in 2020 of 1.6:1, though with decreasing trend over the last few years [40]. One should note that this is despite studies showing that HCV testing is more common among women [41]. The present study also shows that hospitalizations among HCV-infected patients are more frequent in men, especially in the case of 4–7 days LOS subgroup, for which a 1.5:1 ratio was observed. A similar tendency, likely reflecting the sex differences in infection rates, was also noted in studies encompassing other populations, e.g., American [42]. In the case of our research, this difference may be due to several overlapping explanations. First, men in Poland more frequently abuse alcohol and smoke cigarettes [43,44], both of which may have combined adverse effects on the health of patients with HCV infection [45,46,47]. Second, they are also more often obese [48], which is recognized as an independent factor for worse outcomes in HCV infection, including hepatocellular carcinoma [49]. Last but not least, sex differences affect HCV progression and outcome, with men experiencing spontaneous clearance less often [4,50,51] while having higher rates of decompensated cirrhosis and malignant liver tumors, manifested with worse progression [52,53].

Interestingly, we also observed a higher hospitalization rate among urban residents. This again closely reflects the HCV diagnostics since the study encompassing the 2009–2021 period found that 74% of newly diagnosed HCV cases are found in urban areas [52,53,54]. Although this may result from health inequity, i.e., limited access to healthcare and less developed medical infrastructure in rural areas, it is also likely that HCV risk factors are more common in urbanized settings, e.g., tattooing or intravenous use of illicit drugs [54,55]. Moreover, compared to the general population, HCV infection rates are disproportionally higher in men who have sex with men [56,57,58], who tend to live more in urban areas than in rural areas due to greater acceptance and inclusivity, larger gay communities, and better access to resources related to healthcare, including sexual health services [59,60,61]. However, one should note that our observations do not necessarily translate into situations in other world regions, particularly in developing countries. For example, a study conducted in India revealed that most (70%) HCV-infected patients inhabit rural areas [62].

Regardless of sex and place of residency, there was a decreasing trend in the hospitalization rate between 2012 and 2020. One should note that the situation of HCV-infected patients in Poland significantly improved during this period due to the introduction of DAAs. These agents were first registered for use with pegylated interferon in 2011 and later entirely eliminated interferon from therapy in 2015, subsequently leading to high treatment safety and effectiveness [63,64,65]. As shown in the retrospective national study EpiTer-2, accessibility to changing DAA regimens in the era of interferon-free therapy, including the introduction of pangenotypic combinations, resulting in modification of the profile of cirrhotic HCV patients, with a decline in individuals aged over 60 years, suffering from comorbidities, and requiring concomitant medications [62]. Notably, over the course of this study, the flattening of hospitalizations in relation to age was observed, including a decrease in hospitalization of younger patients. It is plausible that this is a result of routine screening of pregnant women for HCV infection, campaigns aiming to provide free laboratory tests, and a publicly funded program for interferon-free therapies in 2015 [63,66]. These efforts resulted in increased diagnosis of HCV infections in Poland, with peaks occurring between 2016 and 2018 depending on sex and age group [66]. At the same time, it is important to stress the need to develop a full-scale national screening program in Poland that will allow for meeting the goal set by WHO to eliminate viral hepatitis as a public health threat by 2030. Screening for anti-HCV should be performed using both classical laboratory methods and rapid cassette tests, which already have a generally recognized diagnostic value. As recommended by the Polish Group of Experts for HCV, such screening should be primarily carried out in primary healthcare facilities, as they provide access to a large number of patients in hospital admission units due to the high percentage of people who could be exposed to the virus in the past, as well as in prisons, which are characterized by the particularly increased incidence of HCV infections [15,67].

On the other hand, it must be highlighted that the lowest hospitalization rate across all LOS classes in the studied 2012–2022 period was noted in years 2020 and 2021, when the COVID-19 pandemic was declared, with subsequent enforcement of sanitary measures and significant organization changes in the healthcare system [68,69]. These changes negatively impacted the availability of care delivered within various medical fields, including hepatology [70,71,72,73]. Data collected from multiple European centers demonstrated that until the end of 2020, the number of HCV outpatient consultations and referrals decreased by 39 and 49%, respectively [74]. Unsurprisingly, in 2022, when (in March) all restrictions related to the COVID-19 pandemic were lifted in Poland [75], a rebound in HCV-related hospitalizations was seen in our study. One should also note that Poland experienced a significant influx of refugees following the war in Ukraine that began in February 2022 [76,77]. Ukraine is characterized by a very high prevalence of HCV infection, estimated at 3.5%, exceeding the European average [78,79,80]. Whether this could, at least to some extent, influence the observed rebound in HCV-related hospitalizations in 2022 in Poland remains unknown from the results of the present study. The previous analysis of HCV infection rates in 2022–2023 did not reveal any apparent increase that could be attributed to the influx of Ukrainian refugees [69].

The present study also reveals the comorbidity characteristics of patients hospitalized with hepatitis C, who most frequently suffered from diseases of the digestive, circulatory, hematological, and immunological systems. Although, based on the analyzed data, it is not possible to understand which of these could be related to the ongoing hepatitis C, it is important to note that other studies revealed that cardiovascular disease, cerebrovascular disease, renal disease, and diabetes are the most common HCV-related comorbidities, increasing hospitalization rates and costs of management of patients with hepatitis C [81]. However, importantly, in our analysis, the comorbidities tended to be less prevalent in patients requiring ≥8 days of hospital stay compared to groups with shorter LOS, indicating they were not the main driving force, highly extending LOS and generating additional costs. Nevertheless, a relatively high incidence of selected comorbidities and mean age of hospitalized patients exceeding 50 years in the analyzed population still indicate that early diagnosis and initiation of treatment is crucial in the management of HCV burden, including decreasing hospitalizations of patients with hepatitis C and associated costs.

Certain limitations of our study need to be stressed. First, the information analyzed provides insight into the characteristics of individuals who received treatment for the disease in the hospital. However, it does not cover individuals who received treatment through outpatient services. Second, this research was performed using diagnostic codes from ICD-10 for HCV patients’ diagnosis confirmation. This approach has limitations due to potential coding inaccuracies and misclassification, which may introduce biases into the results. Third, due to the nature of the Nationwide General Hospital Morbidity Study database, a limitation of this study was the need for more standardization of diagnosis criteria. We relied on clinicians’ professional judgment when entering the diagnostic codes. The quality of routinely collected administrative data has not been verified against a standard reference. Nevertheless, our study presents some advantages concerning its large nationwide sample size, a long observation period, and the inclusion of many variables that can describe the utilization of hospital services during the years 2012–2022.

## 5. Conclusions

This study documents a decline in HCV-related hospitalization rates in Poland, particularly those requiring prolonged stays, likely reflecting the positive impact of enhanced diagnostics, the availability of highly effective DAAs, and national health initiatives aimed at reducing the burden of HCV. Additionally, the data highlight persistent disparities in hospitalization rates among men and urban residents, emphasizing the need for targeted interventions to address these inequities. The observed decline in hospitalizations among younger patients and the flattening of age-related trends suggest that efforts to increase HCV screening and treatment access have been effective in Poland.

However, the study also reveals the significant impact of the COVID-19 pandemic on healthcare delivery, with a notable reduction in hospitalizations in 2020 followed by a rebound as restrictions were lifted. These findings underscore the importance of maintaining robust healthcare services even during global health crises. Going forward, continued political commitment and the implementation of comprehensive national screening programs will be crucial to sustaining progress toward HCV elimination in Poland. Addressing ongoing disparities and ensuring equitable access to care across different demographics and regions remain key priorities in the fight against HCV. An in-depth analysis of HCV co-morbidities is planned that takes into account basic demographic characteristics and possible trends of change, which may provide clinically useful data.

## Figures and Tables

**Table 1:** Number of first-time hospitalizations and rate per 100,000 from 2012 to 2022.

	Year										
	2012	2013	2014	2015	2016	2017	2018	2019	2020	2021	2022
**N**	12,312	7785	7338	6798	7426	5539	5411	4318	1424	1337	1882
**Rate per 100,000**	31.91	20.19	19.04	17.65	19.30	14.23	14.07	11.21	3.72	3.51	4.95

**Table 2:** First-time hospitalizations by sex and place of residence (*n*, % and per 100,000) in 2012–2022 in length of stay (LOS) classes.

LOS (Days)	Characteristics						
	*N*	%	Per 100,000	*n*	%	Per 100,000	Per 100,000 Ratio
Sex	Men			Women			Men /Women
0–3	20,065	63.3	9.84	20,334	68.4	9.34	1.05
4–7	6096	19.2	2.99	4077	13.7	1.87	1.60
≥8	5524	17.4	2.71	5304	17.9	2.44	1.11
Residence	Urban			Rural			Urban /Rural
0–3	29,583	65.9	11.66	10,816	65.6	6.44	1.81
4–7	8191	18.2	3.23	3209	19.5	1.91	1.69
≥8	7149	15.9	2.82	2452	14.9	1.46	1.93

**Table 3:** Hospitalizations per 100,000 in 2012–2022 in different length of stay (LOS) classes.

Year	LOS 0–3	LOS 4–7	LOS ≥8
2012	20.56	6.30	5.04
2013	13.09	3.73	3.37
2014	11.98	4.03	3.03
2015	11.74	3.37	2.54
2016	13.69	3.04	2.57
2017	9.99	2.31	1.93
2018	10.10	2.33	1.64
2019	7.66	1.94	1.62
2020	2.06	0.76	0.90
2021	1.77	0.71	1.03
2022	2.48	1.13	1.33

**Table 4:** Comorbidities in the LOS classes.

ICD-10 Group	LOS 0–3 Days		LOS 4–7 Days		LOS ≥ 8 Days		Total		*p*-Value *
	*n*	%	*n*	%	*N*	%	*n*	%	
Certain infectious and parasitic diseases (A00–B99)	1739	8.42	970	7.32	1734	10.62	4443	8.85	<0.00001
Neoplasms (C00–D48)	1038	5.03	701	5.29	824	5.05	2563	5.10	0.51437
Diseases of the blood and blood-forming organs and certain disorders involving the immune mechanism (D50–D89)	2444	11.84	1888	14.25	1903	11.66	6235	12.42	<0.00001
Endocrine, nutritional and metabolic diseases (E00–E90)	3093	14.98	1466	11.07	1396	8.55	5955	11.86	<0.00001
Mental, behavioral and Neurodevelopmental disorders (F00–F99)	575	2.78	317	2.39	558	3.42	1450	2.89	<0.00001
Diseases of the nervous system (G00–G99)	310	1.50	234	1.77	391	2.40	935	1.86	<0.00001
Diseases of the eye and adnexa and the ear and mastoid process (H00–H95)	121	0.59	55	0.42	47	0.29	223	0.44	0.00009
Diseases of the circulatory system (I00–I99)	4258	20.62	2185	16.50	2617	16.03	9060	18.04	<0.00001
Diseases of the respiratory system (J00–J99)	555	2.69	433	3.27	1025	6.28	2013	4.01	<0.00001
Diseases of the digestive system (K00–K93)	4339	21.01	3499	26.42	3965	24.29	11,803	23.50	<0.00001
Diseases of the skin and subcutaneous tissue (L00–L99)	260	1.26	232	1.75	288	1.76	780	1.55	0.00005
Diseases of the musculoskeletal system and connective tissue (M00–M99)	566	2.74	367	2.77	540	3.31	1473	2.93	0.00253
Diseases of the genitourinary system (N00–N99)	1350	6.54	898	6.78	1035	6.34	3283	6.54	0.31538
**Total**	**20,648**	**100**	**13,245**	**100**	**16,323**	**100**	**50,216**	**100**	

* The significance of the differences applies to the three LOS classes in total.

## Data Availability

Nationwide General Hospital Morbidity Study, National Institute of Public Health NIH-National Research Institute, Warsaw, Poland.
